# A parallelized hybrid genetic algorithm with differential evolution for heat exchanger network retrofit

**DOI:** 10.1016/j.mex.2022.101711

**Published:** 2022-04-26

**Authors:** Jan A. Stampfli, Donald G. Olsen, Beat Wellig, René Hofmann

**Affiliations:** a Lucerne University of Applied Sciences and Arts, Competence Center Thermal Energy Systems and Process Engineering, Technikumstrasse 21, Horw 6048, Switzerland; b TU-Wien, Institute of Energy Systems and Thermodynamics, Getreidemarkt 9/BA, Vienna 1060, Austria

**Keywords:** Heat exchanger network (HEN), Retrofit, Multi-period, Meta-heuristics, Genetic algorithm, Differential evolution, Parallel processing

## Abstract

The challenge of heat exchanger network retrofit is often addressed using deterministic algorithms. However, the complexity of the retrofit problems, combined with multi-period operation, makes it very difficult to find any feasible solution. In contrast, stochastic algorithms are more likely to find feasible solutions in complex solution spaces. This work presents a customized evolutionary based optimization algorithm to address this challenge. The algorithm has two levels, whereby, a genetic algorithm optimizes the topology of the heat exchanger network on the top level. Based on the resulting topology, a differential evolution algorithm optimizes the heat loads of the heat exchangers in each operating period. The following bullet points highlight the customization of the algorithm:

• The advantage of using both algorithms: the genetic algorithm is used for the topology optimization (discrete variables) and the differential evolution for the heat load optimization (continuous variables).
• Penalizing and preserving strategies are used for constraint handling
• The evaluation of the genetic algorithm is parallelized, meaning the differential evolution algorithm is performed on each chromosome parallel on multiple cores.

## Graphical abstract

**Figure:**
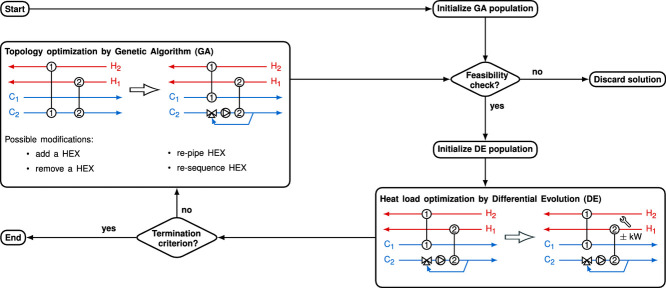

Specification table

**Table:** 

Subject area:	Chemical Engineering, Computer Science
More specific subject area:	Process Integration, Heat exchanger network retrofit
Method name:	Evolutionary based heat exchanger network retrofit for multi-period processes
Name and reference of original method:	n/a
Resource availability:	doi:10.5281/zenodo.4441140

**Table:** 

*Sets*	
C={1⋯j⋯NC}	Set of cold streams
CH={1⋯ch⋯NCH}	Set of heat load chromosomes
CT={1⋯ct⋯NCT}	Set of topology chromosomes
E={1⋯e⋯NE}	Set of heat exchangers
H={1⋯i⋯NH}	Set of hot streams
K={1⋯k⋯NK}	Set of enthalpy stages
GH={1⋯gh⋯NGH}	Set of heat loads generations
GT={1⋯gt⋯NGT}	Set of topology generations
OP={1⋯op⋯NOP}	Set of operating periods
*Parameter*	
a	Admixer existence (boolean)
b	Bypass existence (boolean)
CR	Crossover probability
EAM	Exchanger address matrix / GA chromosome
ex	Heat exchanger existence (boolean)
$F_{p}$	Perturbation factor
f	Fitness function
h	Penalty function
MT	Mutation probability
n	number of infeasible solutions
P	Population
U	trial chromosome
V	donor chromosome
X	Heat load matrix / DE chromosome
Δ	Ineasibility penalty
*Subscripts*	
best	best found solution
init	Initial solution
viol	constraint violations
*Abbreviations*	
DE	Differential evolution
EAM	Exchanger address matrix
GA	Genetic algorithm
HEN	Heat exchanger network
HENS	Heat exchanger network synthesis
HEX	Heat exchanger
HoF	Hall of Fame
TAC	Total annual cost

## Method details

Heat exchanger network synthesis (HENS) is an important tool to design energy efficient production plants in process industry. Furman and Sahinidis [1] showed, that HENS is NP-hard *in the strong sense*. The heat exchanger network retrofit formulation, is an extension of HENS with increased complexity. Complexity is further increased by the additional dimension of operating periods, the possibility to integrate bypasses or admixers, and practical constraints. It is unlikely that deterministic algorithms can provide a feasible solution for such problems on a large-scale. Stochastic algorithms are able to handle such problems. Therefore, a hybrid two-level evolutionary algorithm based on genetic algorithm (GA) and differential evolution (DE) is developed. The concept of GA was first introduced by Holland [2] and further extended by Goldberg [3]. DE was introduced by Storn [4], Storn and Price [5]. In the developed algorithm, a GA handles the topology optimization in the top level and a DE in the sub level is used to optimize the operation parameter.

### Topology optimization using genetic algorithm

**Fig. 1:**
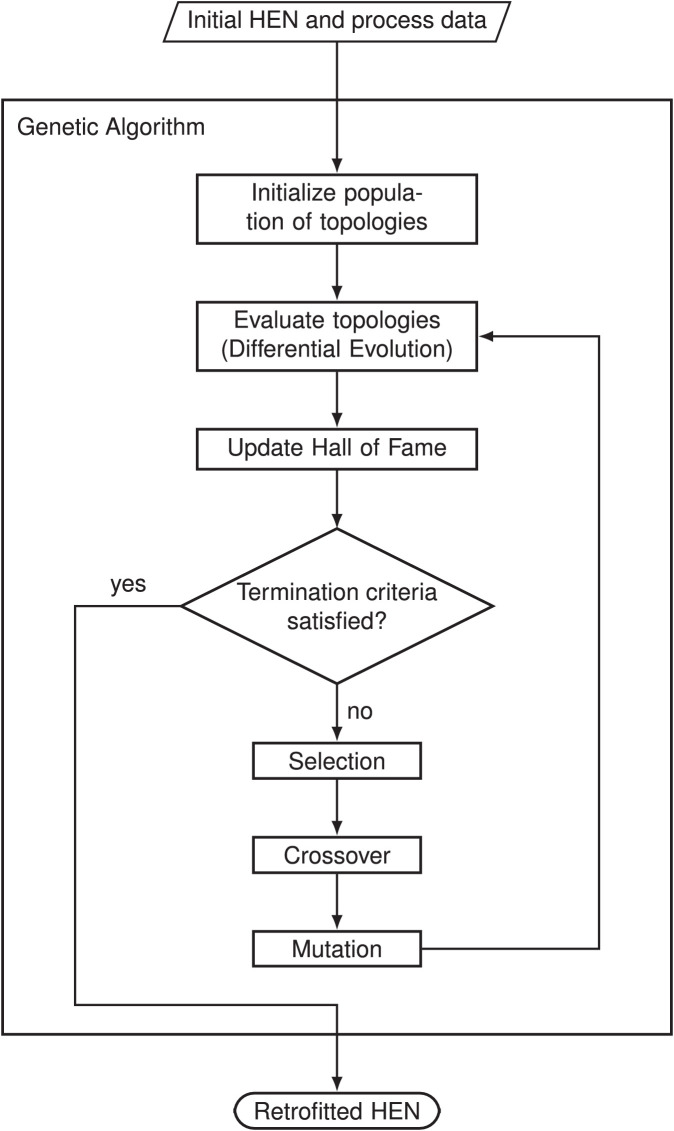
Flowchart of Genetic Algorithm for topology optimization.

In order to define the topology of a HEN, integer values are used to address the position of each heat exchanger (HEX). Genetic algorithms, which are based on the evolution process in nature, are suitable to handle such discrete variables. Thereby, individuals within a population are compared and only the fittest of them survive. During the process, new individuals are generated through the mating of the fittest individuals. Coping errors (mutations) may occur during mating. An overview of the algorithm is shown in Fig. 1. The following sections describe the evolutionary operators in detail.

#### Genetic algorithm - initialization

**Fig. 2:**
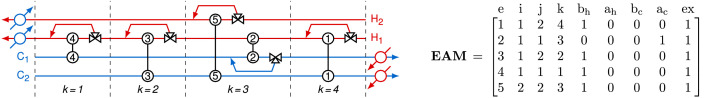
Example heat exchanger network with corresponding exchanger address matrix (bypasses on hot side of HEX 1, 3, 4, and 5; admixer on cold side of HEX 2).

In a first step, a population $P_{t}\;=\;\left\{{\mathrm{EAM}}_{0}\cdots {\mathrm{EAM}}_{NCT}\right\}$ with NCT random individual topologies, hereafter called chromosomes, is initialized. Each of these chromosomes represents a HEN topology and is described using an exchanger address matrix (EAM). An EAM of an example topology is shown in Fig. 2. A chromosome contains the following genes (configurations of a HEX): (e) HEX number, (i) hot stream number, (j) cold stream number, (k) enthalpy stage number, ($b_{h}$) bypass on hot stream, ($a_{h}$) admixer on hot stream, ($b_{c}$) bypass on cold stream, ($a_{c}$) admixer on cold stream, and (ex) existence of the HEX. During the initialization, for heach HEX it is first randomly decided if the HEX exists. For all existing HEXs, a random hot stream, cold stream, and enthalpy stage is defined. To ensure only feasible solution, the preserving constraint handling technique is used (e.g., for the stream number a random value between zero and the number of hot streams NH is selected). Bypass and admixer are not initialized, thus their existence depends on the heat loads. Hence, the need of a mixer and its configuration is determined in the DE.

#### Genetic algorithm - evaluation

After the initialization, each chromosome needs to be evaluated. In order to define the required areas and thus, the resulting cost, the heat loads need to be defined first. The optimal heat loads are found by the DE. To reduce computation time, the penalizing constraint handling technique is used. Thereby, only feasible topologies are optimized using DE. Infeasible solutions are evaluated by a penalty function which is less computationally expensive. The fitness $f_{\text{GA},ct}^{gt}$ of the topology chromosome ct in the topology generation gt is given by

(1) $$f_{\text{GA},\text{ct}}^{gt}\left({\mathrm{EAM}}_{\mathrm{ct}}^{\mathrm{gt}}\right)\;=\;\left\{ \begin{array}{cc} f_{\text{DE},\text{best}}^{\mathrm{gt}}\left({\mathrm{EAM}}_{\mathrm{ct}}^{\mathrm{gt}},X_{\mathrm{ct},\mathrm{best}}^{\mathrm{gt}}\right) & \text{if}\;n_{\text{GA},\text{viol}}^{\text{gt}}=0\\ \frac{1}{h_{\text{GA}}^{\mathrm{gt}}\left(n_{\text{GA},\text{viol}}^{\mathrm{gt}}\right)} & \text{otherwise} \end{array}\right.$$

whereby, $f_{\text{DE},\text{best}}^{gt}$ is the fitness of the DE in function of the EAM and the heat loads of the best DE solution $X_{ct,best}^{gt}$. The distance of each violated constraint c is given by $n_{\text{GA},\text{viol},c}^{gt}$. For infeasible topologies, the fitness is defined by reciprocal value of the penalty function, given by

(2) $$h_{\text{GA}}^{gt}\left(n_{\text{GA},\text{viol}}^{gt}\right)\;=\;\Delta \;+\;\underset{n_{\text{GA},\text{viol}}^{gt}}{\underset{︸}{\sum_{c\;\in \;C_{\text{GA}}}{\left(0-n_{viol,c}^{gt}\right)}^{2}}}\;\text{with}\;\Delta \;>>\;{\mathrm{TAC}}_{\text{init}}$$

whereby $C_{\text{GA}}$ is the set of all GA constraints. A constant value Δ, which is larger than the initial total annual cost of the existing process, is added to the sum of the squared distances to the feasible region.

#### Genetic algorithm - selection

To choose the parent chromosomes for mating, tournament selection is performed. This means that the fittest out of a given number of randomly selected chromosomes is chosen.

#### Genetic algorithm - crossover

With a crossover probability of $CR_{\text{GA}}$, the parents mate. Thereby, a one-point crossover is performed. Both parent chromosomes are cut below a random selected HEX number. The HEXs above the cut are swapped between the two chromosomes. The two resulting children chromosomes replace the parent chromosomes and are evaluated.

#### Genetic algorithm - mutation

In nature, during the mating process in the crossover, coping errors (mutations) may occur. In the algorithm, with a mutation probability of $MT_{\text{GA}}$, for each allele (a single scalar value within a gene), a mutation occurs. For the mutation in the existence of a HEX gene (ex), a random bit flip is performed. In the genes (i), (j), and (k), the value is changed within the upper and lower boundary (e.g., for (i) the number of existent hot streams) of the gene using an uniform distribution. It is ensured by using the preserving strategy, none of these values exceed their boundaries.

#### Genetic algorithm - next generation

After the application of the evolutionary operators (selection, crossover, and mutation), the new generated topologies are evaluated and the population is updated by replacing the parent chromosome with the new children chromosomes for the next generation. In order to keep track of the best solutions, a Hall of Fame (HoF) list is created. This list contains the current best solutions. If in the evaluation step a fitter solution is found, the HoF list is updated. With this additional feature, the flexibility of the algorithm in use is increased as the engineer is now able to choose between the most promising solutions. The algorithm is terminated as soon the maximal number of topology generations NGT is reached.

### Heat load optimization using differential evolution

**Fig. 3:**
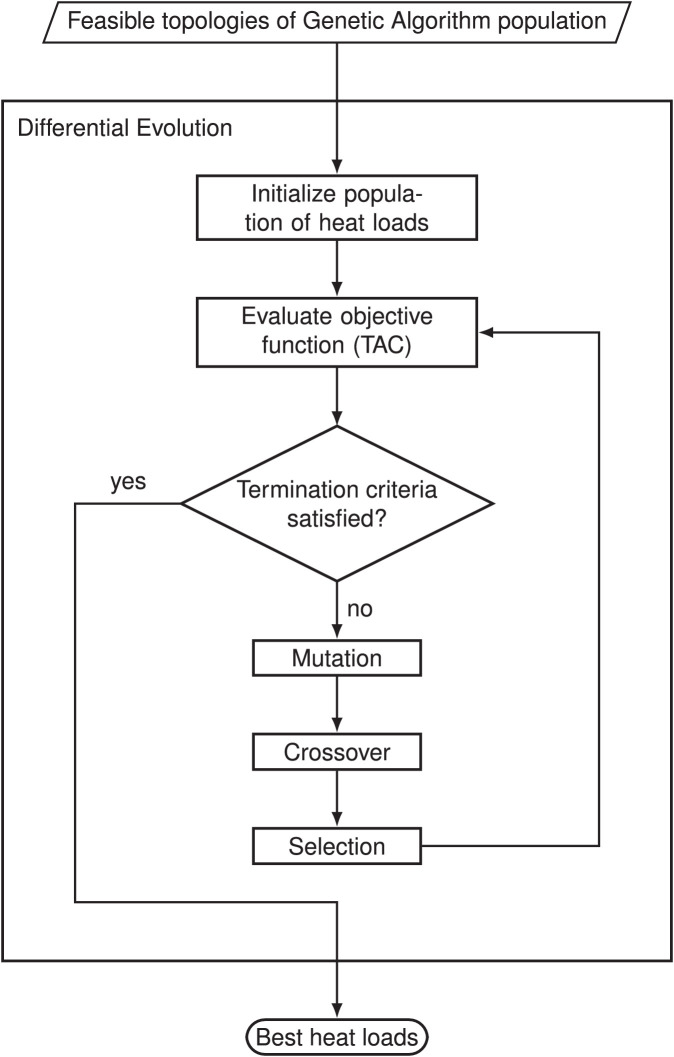
Flowchart of Differential Evolution for operation parameter optimization.

In contrast to the topology optimization, the heat loads are continuous variables with upper and lower bounds. Differential evolution algorithms are best to deal with the continuous variables. Compared to deterministic algorithms such as gradient descent, DE does not require the model to be differentiable. DE algorithms use the same concepts of evolution as GAs. However, the order of the evolutionary operators is reversed. There are different options for the DE configuration. In this case, the standard configuration *DE/rand/1/bin* is used. This means that the individuals for mutation are selected randomly, only one difference for perturbation ($F_{P}$: perturbation factor) is considered, and a binomial crossover is performed. In Fig. 3, an overview of the algorithm is shown. The following sections describe the algorithm in detail.

#### Differential evolution - initialization

For each feasible topology from the GA, a population $P_{h}\;=\;\left\{X_{0}\cdots X_{NCH}\right\}$ with NCH random heat load chromosomes is initialized. Each of these chromosomes consists of all the heat loads of each heat exchanger in every *OP*, resulting in a two-dimensional array. Chromosome ch is given by

(3) $$X_{\mathrm{ch}}\;=\left({\dot{Q}}_{e}^{op}\right)$$

whereby the heat load is an array of the size NE×NOP. The initialization of the heat loads of existing HEXs ${\dot{Q}}_{ch,e}^{op}$ is constraint by a minimal user defined value and a maximal value given by the enthalpy differences of the connected streams (case study dependent value).

#### Differential evolution - evaluation

For the evaluation of the population of heat loads, the fitness of each chromosome needs to be determined. Infeasible solutions are evaluated by a penalty function. In each generation gh the fitness $f_{\text{DE},ch}^{gh}$ is given by

$$f_{\text{DE},ch}^{gh}\left({\mathrm{EAM}}_{ct}^{gt},X_{ch}^{gh}\right)\;=\;\left\{ \begin{array}{cc} \frac{1}{TAC({\mathrm{EAM}}_{ct}^{gt},X_{ch}^{gh})} & \text{if}\;n_{\text{DE},viol}^{gh}=0\;\;\;\;\;\;\;\;\;\left(4\right)\\ \frac{1}{h_{\text{DE}}^{gh}\left(n_{\text{DE},viol}^{gh}\right)} & \text{otherwise.}\;\;\;\;\;\;\;\;\;\;\;\;\left(5\right) \end{array}\right.$$

whereby, the TAC is the total annual cost of the current topology and heat loads. TAC consists the yearly operating cost and the investment cost for the retrofit. The detailed model for calculating the TAC is formulated in the corresponding research paper [6]. In order to minimize the TAC, the reciprocal value represents the fitness of a chromosome. Each distance of a heat load constraint violation is given by $n_{\text{DE},viol,c}^{gh}$. For infeasible operation parameter, the fitness is defined by reciprocal value of the penalty function, given by

(6) $$h_{\text{DE}}^{gh}\left(n_{\text{DE},viol}^{gh}\right)\;=\;\Delta \;+\;\underset{n_{\text{DE},viol}^{gh}}{\underset{︸}{\sum_{c\;\in \;C_{\text{DE}}}{\left(0-n_{viol,c}\right)}^{2}}}\;\text{with}\;\Delta \;>>\;TAC_{init}$$

whereby $C_{\text{DE}}$ is the set of all DE constraints. A constant value Δ which is larger than the initial TAC is added to the sum of the squared distances to the feasible region.

#### Differential evolution - mutation

The first evolutionary operation in a DE is mutation. Thereby, a three non-equal chromosomes $X_{r1}^{gh},X_{r2}^{gh},X_{r3}^{gh}$ ($r_{1}\;\neq \;r_{2}\;\neq \;r_{3}$) of the current generation gh are selected randomly. A new donor chromosome is generated by

(7) $$V_{ch}^{gh}\;=\;X_{r1}^{gh}+F_{P}\;\left(X_{r2}^{gh}-X_{r3}^{gh}\right)$$

whereby one difference for perturbation is used and weighted with the perturbation factor $F_{P}\;\in \;\left[0,2\right]$. After the mutation, for all infeasible heat loads a new random value within the boundaries is assigned.

#### Differential evolution - crossover

In the crossover operator, a trial chromosome $U_{ch}^{gh}$ is generated by

(8) $$\begin{array}{c} u_{ch,e}^{gh,oc}\;=\;\left\{ \begin{array}{cc} v_{ch,e}^{gh,oc}, & r^{gh}<CR_{\text{DE}}\;\vee \;p\;=\;r\\ x_{ch,e}^{gh,oc}, & \text{otherwise} \end{array}\right. \end{array}$$

whereby, $r^{gh}∼U\left(0,1\right)$ has a uniform distribution. With a probability of $CR_{\text{DE}}$ a crossover is performed. To ensure at least one crossover per operating parameter, a random index within the chromosome is chosen for which crossover is always performed.

#### Differential evolution - selection

In the selection operator, the new created trial chromosome is evaluated. To determine the new target chromosome for the next generation gh+1 with

(9) $$\begin{array}{cc} X_{ch}^{gh+1}\; & =\;\left\{ \begin{array}{c} U_{ch}^{gh},\;\text{if}\;f\left(U_{ch}^{gh}\right)\;>\;f\left(X_{ch}^{gh}\right)\\ X_{ch}^{gh},\;\text{otherwise,} \end{array}\right. \end{array}$$

a simple greedy selection is performed.

#### Differential evolution - next generation

For each generation, the evolutionary operators (mutation, crossover, and selection) are executed till one of the termination criteria is fulfilled. The first termination criterion is satisfied when the maximal number of heat load generations NGH is reached. The second termination criterion is reached when the number of consecutive generations without improvement of the fitness exceeds its limit.

### Implementation and parallelization

The algorithm is implemented in Python 3.8.2 using the library *DEAP - Distributed Evolutionary Algorithms in Python* [7]. Evolutionary algorithms are predestined for parallel computing as they work with populations of chromosomes which can be evaluated separately by distributing them among multiple processors. Therefore, all feasible GA chromosomes are distributed to multiple processors on which the heat loads are optimized using DE. The source code of the algorithm is published under an open-source license [8].

## Conflict of Interest

The Authors confirm that there are no conflicts of interest.
